# Warming in the Agulhas Region during the Global Surface Warming Acceleration and Slowdown

**DOI:** 10.1038/s41598-018-31755-1

**Published:** 2018-09-07

**Authors:** Lu Han, Xiao-Hai Yan

**Affiliations:** 1 College of Earth, Ocean and the Environment, University of Delaware, Newark, USA; 2 University of Delaware and Xiamen University’s Joint Institute for Coastal Research and Management, Newark, USA

## Abstract

The Agulhas Region gains more heat during the global surface warming slowdown than acceleration period. Yet, mechanisms that cause excessive heat accumulation in this region remain largely unknown. We investigate the underlying physical processes and examine their influence on ocean heat changes in the last three decades. Heave is found to drive the increasing ocean heat content in the last three decades whereas elevated heat accumulation rate in slowdown compared than acceleration period is mainly attributed to spice. During the acceleration period, pure heaving of Subtropical Mode Water induced by wind stress change and pure warming caused by heat flux, leading to a strong heave component and relatively weak spice, drive the increases in ocean heat content. During the slowdown period, increasing salinity strengthens the spice, resulting in a higher heat accumulation rate compared to the acceleration period.

## Introduction

Anthropogenic CO_2_ emission continuously increases greenhouse gases (GHG) and contributes to elevated heat content in the earth system. The annual mean atmospheric concentration of CO_2_ observed in Mauna Loa, Hawaii, increased from about 315 ppm in the 1960s to 396 ppm in 2013^1^. However, following the strong El Niño event in 1998, global mean surface temperature (GMST) increased at a much slower rate than that of the latter half of the 20th Century. The Inter-Governmental Panel on Climate Change (IPCC) Fifth Assessment Report (AR5) reported that during this time period, the warming rate has stalled 0.05 °C/decade, compared to 0.12 °C/decade since 1951, and referred this period as the “global warming hiatus”^1^. This topic has raised many debates. Karl *et al*. argued that the “hiatus” stems from data biases and is not a real signal^2^. Recently a panel of scientists have reached to a consensus and confirmed the slowdown of GMST rising rate, but they also pointed out that “the phenomenon is only a surface characteristic that does not represent a slowdown in warming of the climate system but rather is an energy redistribution within the oceans”^3^. Therefore, this period (from 1998 to 2013) is corrected as “global surface warming slowdown” instead of “global warming hiatus”.

**Figure 1:**
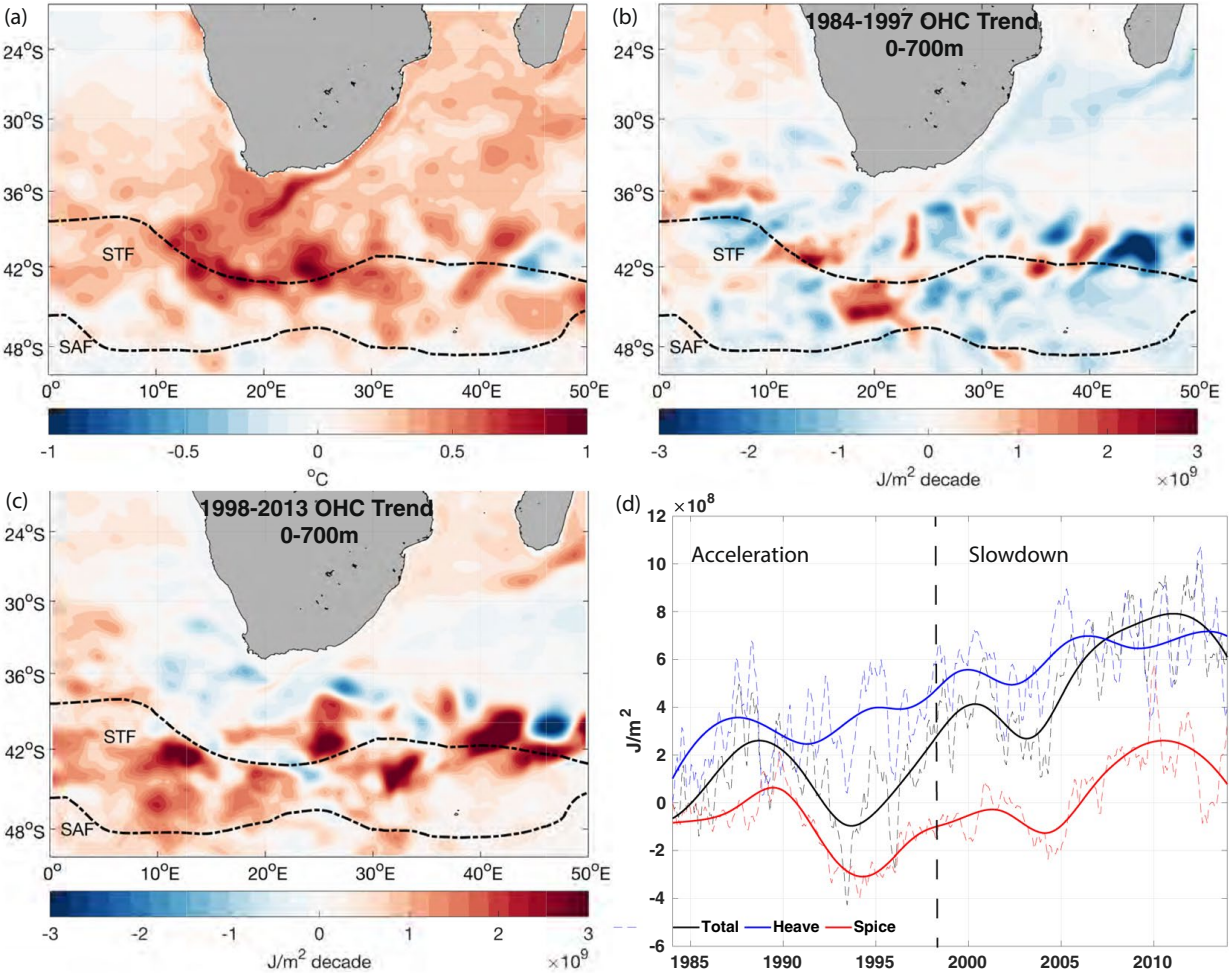
(**a**) Temperature difference between the two time periods (Mean temperature of [1998–2013] minus that of [1984–1997]). (**b**) OHC trend at the upper 700 m layer between 1984 and 1997. (**c)** OHC trend at the upper 700 m layer between 1998 and 2013. The two black dash lines in (**a**,**b**,**c**) are the subtropical front (STF) and the subantarctic front (SAF), respectively^24^. (**d**) The averaged OHC at the Agulhas Region and its heaving and spice components. The dash lines are the original monthly data. The solid lines are their IMF5-7 plus long-term nonlinear trend. Black is the total OHC; blue is the heave component; and red is the spice component.

The Agulhas Region (AR) (Fig. 1a) is one of the fastest warming regions in the world ocean in the last few decades^4,5^ and can be one of the oceanic sinks for the excess heat due to heat redistribution^3^. This region consists of the Agulhas Current, the western boundary current of the Indian Subtropical Gyre, retroflection, where the Agulhas Current turns anticlockwise after separating from the coast, and the Return Current, which flows back to the Indian Ocean^6^. The abrupt turn interacts with bathymetry and generates rings, mesoscale eddies, and filaments, leaking the warm and saline water into the neighboring Atlantic Ocean, known as the Agulhas Leakage, and feeding the upper arm of the Atlantic Meridional Overturning Circulation (AMOC)^7–9^. The Agulhas Current and Agulhas leakage serves as the pathway of the heat transport between the Indian Ocean and the South Atlantic Ocean and may play a significant role in heat redistribution globally^10^.

A key to examining Agulhas Region’s influence on global heat redistribution during the global surface warming slowdown is a basic understanding of warming mechanisms in this region. We decompose the temperature change over the previous three decades, including 15-year surface warming acceleration and 15-year surface warming slowdown, into two components, “heave” and “spice”. Heave characterizes the temperature change due to the vertical movement of isopycnals whereas spice determines the temperature change along the isopycnals^5^. Our results demonstrated heave component is more dominant in the Agulhas Region and identified the difference in warming mechanisms between the global surface warming acceleration and slowdown period.

## Results

Agulhas Region (AR) covers most of the Greater Agulhas System, located in the domain delimited by 10°E to 35°E and 36°S to 45°S^11^, including the south end of the Agulhas Current, the retroflection, the Return Current and its adjacent subtropical front. Sea surface temperature data and two reanalysis data sets (ORAP5 and ORAS4), were analyzed to study the heat distribution and warming mechanism from 1984 to 2013. Results from the two reanalysis datasets are highly consistent (Fig. S1); therefore, only results from ORAP5 are presented due to its higher spatial resolution (ORAP5: 0.25^o^ × 0.25^o^ × 75 vertical levels vs. ORAS4: 1^o^ × 1^o^ × 42 vertical levels). To compare the heat distribution pattern between the global surface warming acceleration and slowdown period, data from 1984–1997 is used to represent global warming acceleration period and 1998–2013 for slowdown.

The AR shows a strong warming trend at the surface in the last three decades^11^. The strongest surface warming signal is detected at the retroflection, Return current, and its adjacent subtropical frontal region (Fig. 1a). As sea surface temperature only captures surface signal, ocean heat content (OHC) is further used to investigate interior processes in the AR. In the last three decades, the increase of OHC anomalies is mainly detected at 0–700 m (Figs S1, S2). The rate of heat accumulation is shown to be much higher during the slowdown than the acceleration period (Fig. 1b,c), consistent with previous findings that more heat sinks into the interior ocean during the slowdown^3,12–14^.

In the upper 200 m layer, diabatic processes such as seasonality and mixing are dominant in the AR, resulting in substantial residue from heat decomposition^15^. Thus, in this study, we focus on the 200–700 m layer to investigate the heat evolution and distribution in the AR. To obtain a general trend of heat content changes induced by heave and spice, Ensemble Empirical Mode Decomposition (EEMD) is applied to all the three signals (total OHC, heave, and spice) (Fig. S3), decomposed into intrinsic mode functions (IMF) with different time scales^16^. In this study, each EEMD mode passes the significant test determined by calculating the spread function for the 99% and 95% confidence limit levels^17^. After removing the seasonal and annual viability, decadal variability plus the long-term nonlinear trend show an increasing pattern for heat content changes induced by heave during the three decades, while the OHC and spice component are observed to have an increase only in the slowdown period with high variability during the acceleration period. Moreover, OHC change induced by heaving process is much higher than that induced by spice (Fig. 1d). The mean value of the heave component in the AR is 4.7 × 10^8^ J/m^2^, while that of spice component is −2.6 × 10^7^ J/m^2^, suggestive that heave is a major contributor to the elevated heat accumulation in interior ocean, which is also consistent with previous findings that isopycnals in the AR have one of the largest deepening rates globally^5^. Noticeably, IMFs5-7 for spice display a similar pattern with that for the total OHC, which indicates that the decadal variabilities of the total OHC can be mainly attributed to the spice processes (Fig. 1d).

**Figure 2:**
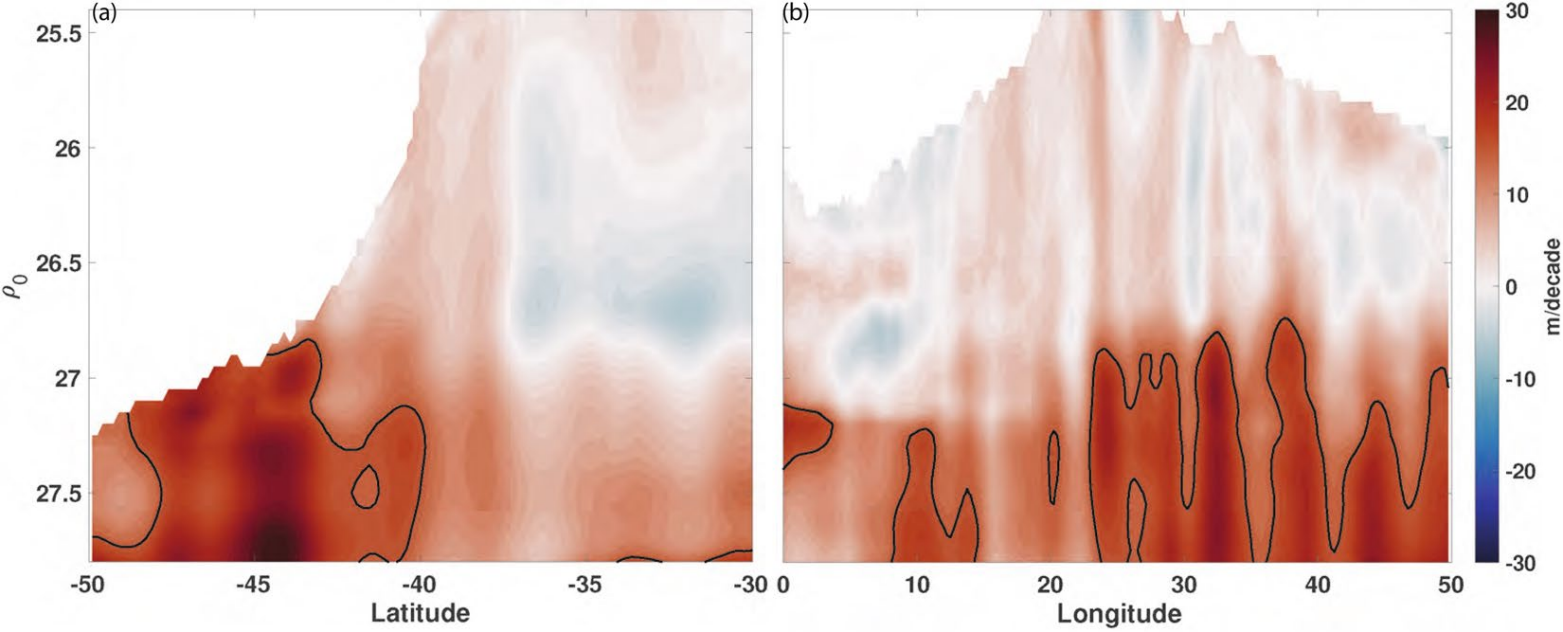
(**a**) Zonal, (**b**) Meridional average of deepening/shoaling trends of the potential density surfaces for the Agulhas Region in the last three decades (m/decade; positive downward). The black contour is 15 m/decade.

**Figure 3:**
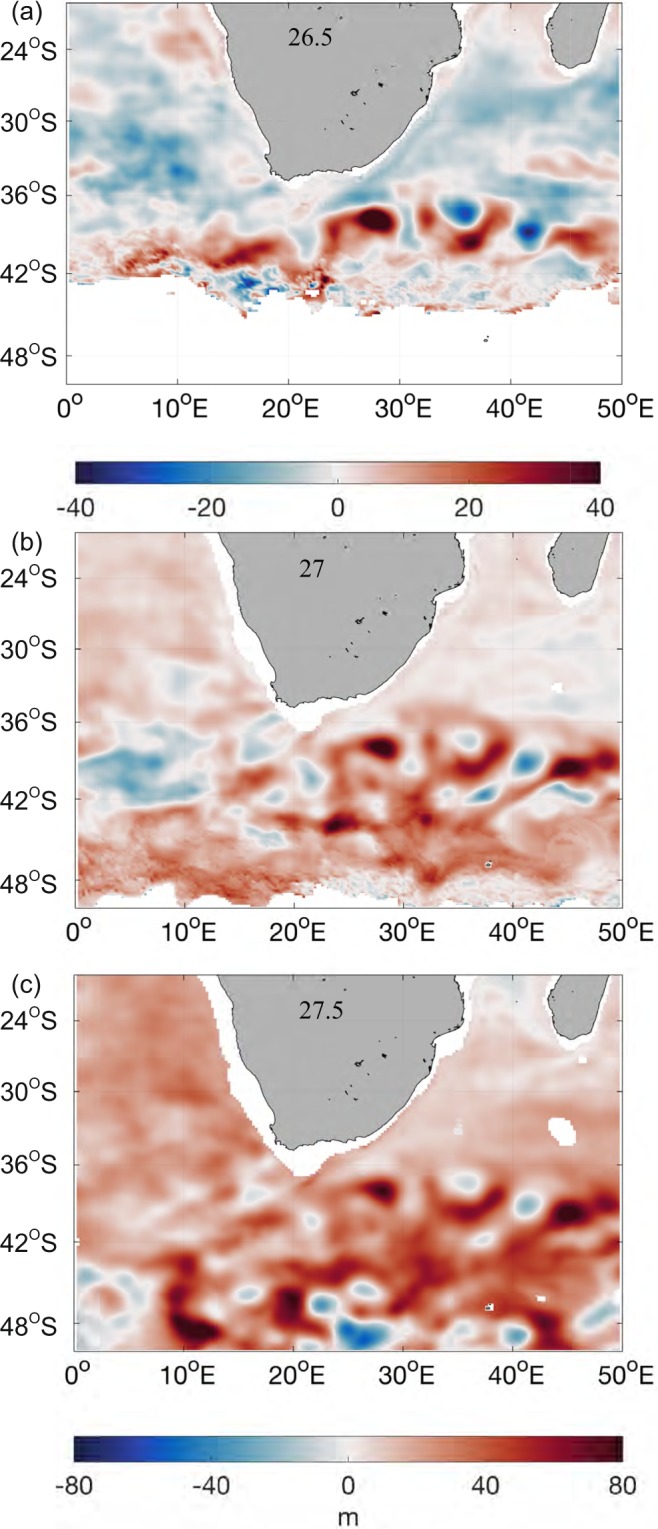
Deepening/shoaling change of the potential density surfaces (**a**) 26.5, (**b**) 27, (**c**) 27.5 from 1984–1998 to 1999–2013 at the Agulhas Region. (**b**) and (**c**) share the same colorbar.

Heat content changes in ocean interior caused by heave are shown to vary at different density surface (Fig. 2). Strong isopycnal deepening, over 15 m/decade, is observed in Antarctic Intermediate Water (AAIM) and Central Water (CW) (σ = 26.8–27.8), at the deep layer of the Return Current and upper layer of its adjacent subtropical frontal region (Figs 2, S4). Noticeably, Subtropical Mode Water (STMW: δ ≤ 26.5) in Agulhas Current displays a shoaling trend (Fig. 3), which indicates that heave’s impact changes in both horizontal and vertical dimensions. To further identify the water mass that contributes the most to heat content changes induced by heave, isopycnals with σ at 26.5, 27, 27.5 are chosen to examine their various influence on ocean heat content. Compared to the previous acceleration period, density surface at 26.5 deepens around 40 meters in the Return Current and shoals in the Agulhas Current during the slowdown period. In contrast to the strong spatial variability detected in water mass at 26.5, isopycnals at 27 and 27.5 present a more uniform deepening signal with twice the deepening rate on average. Häkkinen *et al*.^5^ claimed that the strongest deepening happens at isopycnal at 27 in the southern hemisphere. However, our study implies that the isopycnal of 27.5’s deepening is stronger than that of 27 in the AR.

**Figure 4:**
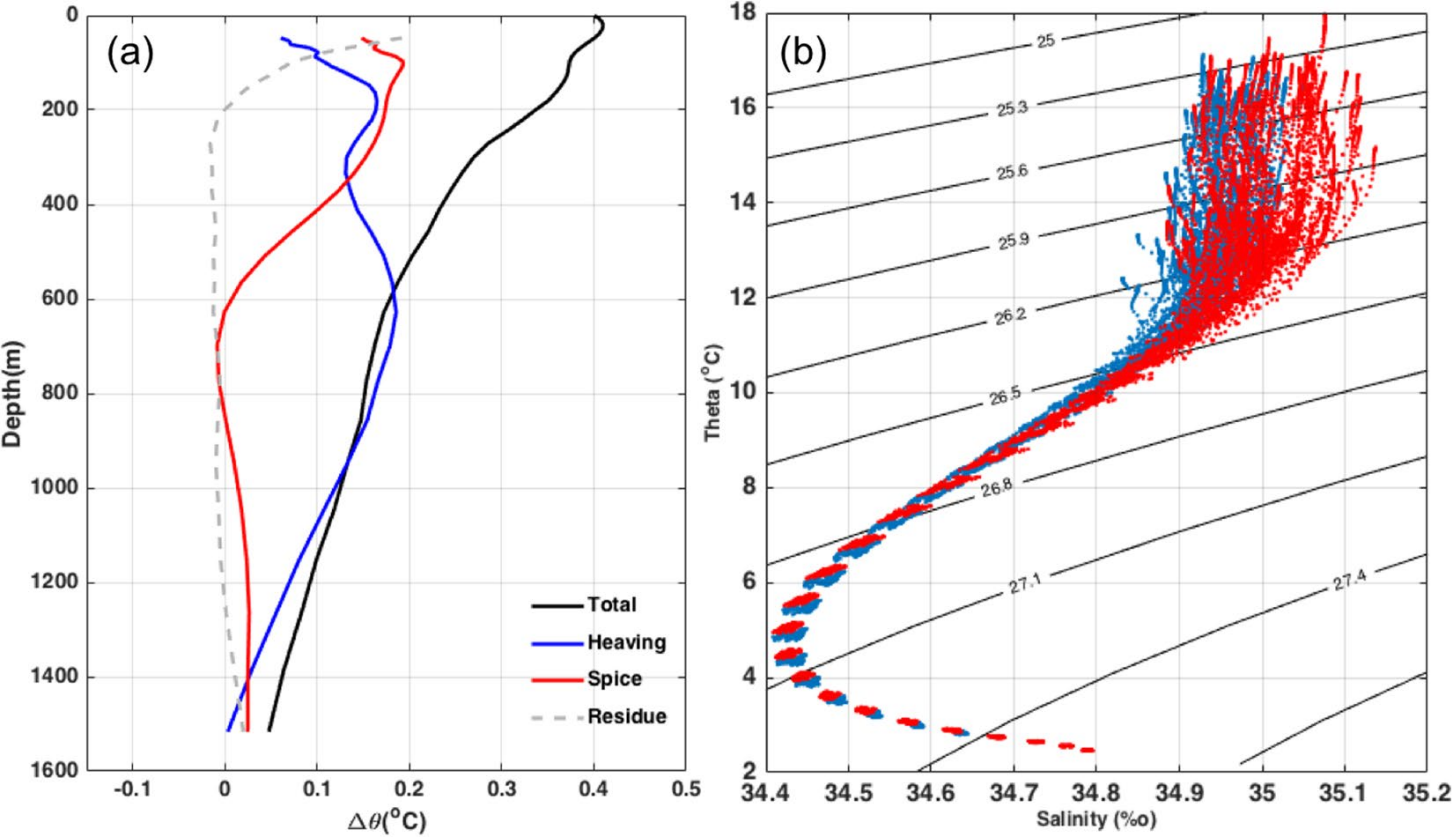
(**a**) Total potential temperature change (^o^C) from the acceleration (1984–1998) to the slowdown (1999–2013) divided into heaving and spice in the Agulhas Region. The black line is the total temperature change; the blue line is the heaving component; the red line is the spice component; and the gray dash line is the residue of total OHC minus the heave and spice components. (**b**) Averaged T-S Diagram at the Agulhas Region during the two periods. Blue dots represent the temperature and salinity during the acceleration period (1984–1997) and red dots represent the slowdown period (1998–2013).

To obtain a higher resolution of heat content change across the water column, vertical profiles of water temperature change is examined to assess the relative contribution of heave and spice at different depth in the AR. The total temperature difference between the slowdown and acceleration period decreases with depth (Fig. 4a). Vertical mixing, inferred by the residue of the decomposition, can be important at the surface while temperature changes below 200 m are mainly attributed to heave and spice. Heave has a strong impact on temperature variations across the whole water column whereas spicing component decreases with depth, which is similar to the vertical profile of total temperature difference. The high temperature changes caused by spice indicates the elevated heat content accumulation in slowdown compared to acceleration period is mainly caused by elevated spicing.

## Conclusion and Discussion

The Agulhas Region (AR) is one of the fastest warming regions in the world ocean in the last few decades^4,5^. Heat accumulates at a faster rate during the slowdown than the acceleration period. Therefore, it is considered as one of the possible oceanic heat sinks. Despite its essential role in global heat redistribution, a basic understanding of the mechanisms leading to its excessive heat accumulation is still lacking. This research decomposes temperature changes into heave and spice, which are two major adiabatic physical processes affecting ocean heat content, to assess their impacts on excessive heat accumulation in the AR during the slowdown period. Increasing heat content is mainly caused by heave at the deep layer (200–700 m) whereas the elevated heat accumulation rate observed in slowdown can be mainly attributed to stronger spice. Heave is found to increase in a monotonic fashion while more decadal variabilities are observed for the spice component. The total OHC also shows a strong decadal variability, indicating that decadal variability in OHC can be largely attributed to spicing processes. Noticeably, heave displays spatial variations both horizontally and vertically, further highlighting the complex nature of adiabatic physical processes in the AR.

**Figure 5:**
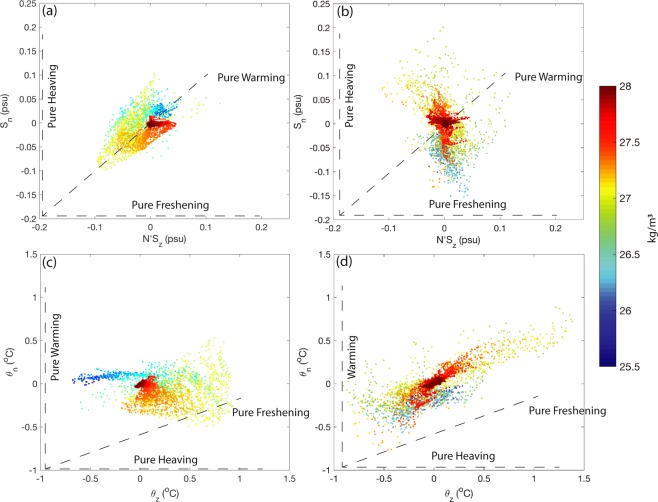
Hodograph of (**a**,**b**) the spice and heave components of salinity change trend, (**c**,**d**) temperature change trend along pressure surfaces and neutral surfaces. Trends during the acceleration periods are in (**a**) and (**c**), while trends during the slowdown period in (**b**) and (**d**). Colored dots parallel to the three dash lines labeled with Pure Warming, Pure Freshening and Pure Heaving represent along which are the contribution of pure warming, pure freshening and pure heaving, respectively. The color represents the density of neutral surfaces.

In ventilated thermocline theory, the surface conditions can be advected into the interior along isopycnals^18^. Following previous methodology^15^, we investigate three possible surface processes contributing to the ocean interior warming, pure warming due to the heat flux from the atmosphere to the ocean, pure freshening caused by the changes of evaporation and precipitation, and pure heaving stimulated by wind stress change. Water mass change (pure warming or pure freshening) induced heaving can counteract or reinforce wind stress change induced heaving (pure heaving). Pairs of temperature or salinity change trend at pressure surfaces (*θ*_*z*_, *S*_*z*_), its heave (*N*′*θ*_*z*_, N′S_z_) and spice (*θ*_*n*_, *S*_*n*_) components are compared during the acceleration and slowdown periods (Fig. 5). In the pure warming scenario, *S*_*z*_ = 0, which requires the salinity changes on a neutral surface to be balanced by the vertical movement of the neutral surface (*S*_*n*_ = N′S_z_). Likewise, in the pure freshening scenario, the temperature changes at certain depth should equal the changes on the neutral surface (*θ*_*z*_ = *θ*_*n*_). And pure heaving scenario involves no temperature or salinity change along the neutral surface (*θ*_*n*_ = 0, *S*_*n*_ = 0). During the acceleration period, the correlation coefficient between *S*_*n*_ and N′S_z_ is 0.5358 (Fig. 5a), which indicates that increase of heat flux is one of the dominant warming mechanisms. Moreover, no temperature change of the STMW along the neutral surfaces (Fig. 5c) suggests that STMW experienced wind driven heaving during the acceleration period. However, during the slowdown period, STMW is saltier than that during the acceleration period (Fig. 4b), and the warming is governed by salinity change and pure heaving of most of the water column. A correlation coefficient of 0.7962 was found between *θ*_*z*_ and *θ*_*n*_(Fig. 5d), and S_n_ falls to near zero (Fig. 5b) with a correlation coefficient of 0.6406 between *S*_*z*_ and N′S_z_. The salinity change trend is positive at where *θ*_*z*_ and *θ*_*n*_ are best correlated with each other, suggestive that it is more of a salting scenario during the slowdown period rather than freshening. Pure warming and pure freshening can lead to negative spice and positive heave^5^. During the acceleration period, pure heaving of STMW induced by wind stress change combines heat flux into the ocean, leading to the fast increase of the heave component and relatively weak spice. Whereas, during the slowdown period, strong pure heaving is offset by the increasing salinity, which can also lead to a larger spice comparing to the former period.

## Data and Methods

We followed the decomposition method introduced by Bindoff and Mcdougall^15^ and updated by Häkkinen *et al*.^5^ and Zhang and Yan^19^.

$$\frac{d\theta }{dt}{|}_{z}=\frac{d\theta }{dt}{|}_{n}-\frac{dz}{dt}{|}_{n}\frac{d\theta }{dz}+Residue$$

$$\frac{dS}{dt}{|}_{z}=\frac{dS}{dt}{|}_{n}-\frac{dz}{dt}{|}_{n}\frac{dS}{dz}+Residue$$

As shown in the equation, the potential temperature changes at a certain depth (${\theta }_{z}=d\theta /dt{|}_{z}$) are divided into a spice component (${\theta }_{n}=d\theta /dt{|}_{n}$), a heave component (${\rm{N}}^{\prime} {{\rm{\theta }}}_{{\rm{z}}}={\rm{dz}}/{\rm{dt}}{|}_{n}d{\rm{\theta }}/{\rm{dz}}$), and residue, which is due to vertical mixing. In a well-stratified environment, the residue should be near zero. Heave is the temperature change due to the movement of a neutral density surface, while spice is the change along one. Neutral density surface here is an isopycnal with same potential density to a nearly continuously varying reference pressure, an approximation to the isentropic surface^20^. For the water parcel to move along and stay on the same isopycnal, the temperature change must come along with salinity change. Thus, the “spice” component is also known as water mass change. Total ocean heat content (OHC) change and its heave and spice components are computed by integrating potential temperature changes times the volume, density, and heat capacity of seawater.

Ensemble Empirical Mode Decomposition (EEMD) is used to the OHC change, its heave component and spice component calculated from ORAP5 reanalysis data. EEMD is a recent method for decomposing a time series into intrinsic mode functions (IMF) along with a trend, which can be applied to nonstationary and nonlinear data^16,21^. The significance of each resulting intrinsic mode functions (IMF) in this study was determined by calculating the spread function for the 99% and 95% confidence limit levels^17^. All the IMFs in this study pass the significant test.

## Electronic supplementary material

Supplementary Information

## Data Availability

We used the Ocean ReAnalysis Pilot 5 (ORAP5)^22^ and Ocean Reanalysis System 4 (ORAS4)^23^ data from 1984 to 2013 to investigate the temperature and salinity (TS) structure and calculate the heat ocean content (OHC) at the Agulhas Region. The datasets analyzed during the current study are available in the CMEMS repository, ftp://rancmems.mercator-ocean.fr/Core/GLOBAL_REANALYSIS_PHYS_001_017 with a CMEMS account required and http://icdc.cen.uni-hamburg.de/thredds/aggregationOras4Catalog.html, respectively.
