# Supplementary material for: Warming in the Agulhas Region during the Global Surface Warming Acceleration and Slowdown
Source: Sci Rep. 2018 Sep 7;8:13452. doi: 10.1038/s41598-018-31755-1 (PMC6128912; doi:10.1038/s41598-018-31755-1)
Supplement: Supplementary file 1 — Supplementary Information [file 41598_2018_31755_MOESM1_ESM.docx]

**Warming in the Agulhas Region during the Global Surface Warming Acceleration and Slowdown**

**Lu Han**1**, Xiao-Hai Yan**1,2,*

1College of Earth, Ocean and the Environment, University of Delaware

2Joint Institute for Coastal Research and Management, University of Delaware/Xiamen University, USA/China

*[xiaohai@udel.edu](mailto:xiaohai@udel.edu)

# Supplementary Information

Fig. s1: Annual regional averaged OHC in the Agulhas Region computed from ORAP5 (solid lines) and ORAS4 (dash lines) reanalysis data. Red, blue and gray lines represent for upper 200m, 700m, 1500m respectively.

Fig. s2: OHC trend during the acceleration and slowdown periods at different layers. The unit is J/m^3^Decade, which is the area averaged OHC trend normalized by the depth for better comparison.


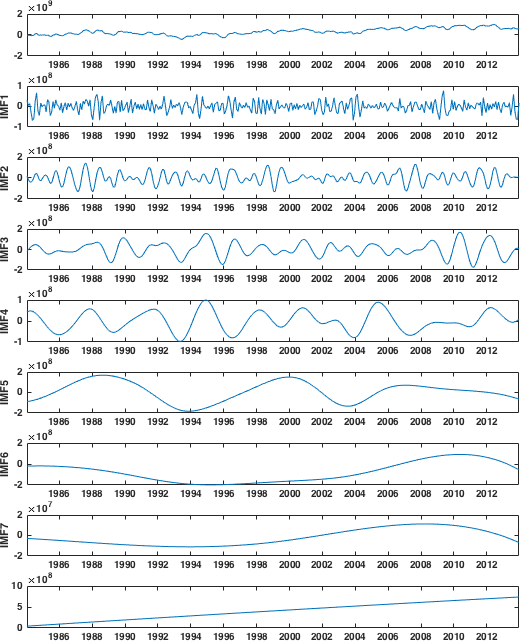

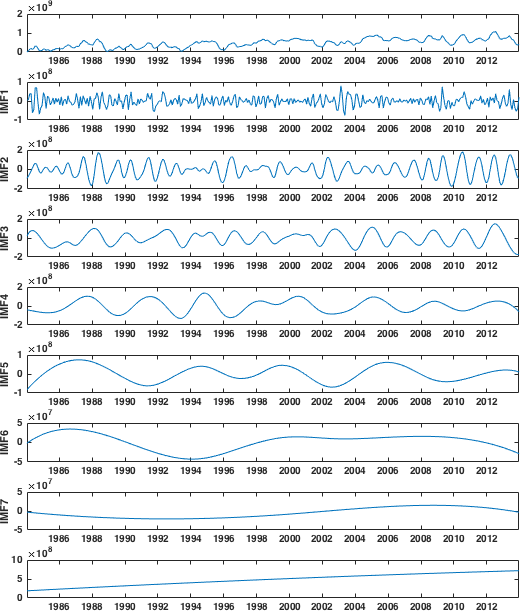

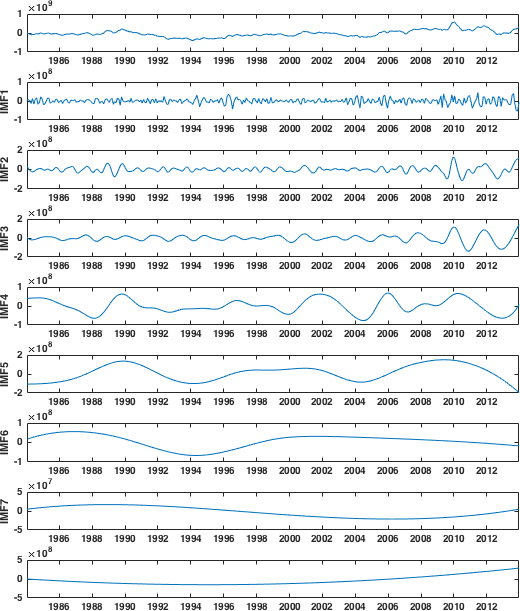


(a)

(b)

(c)

Fig. 3s: EEMD of (a) the total OHC (OHC) (200-700m), (b) heave component, and (c) spice component at the Agulhas region. IMFs with seasonal to decadal time scales, and the residual of the decomposed signal. The IMFs are plotted in order from high to low frequency. The x axis is time in years. The y axis has units of J/m^2^. The IMFs have the mean removed, while the residual preserve the mean location.

Fig. s4: (a) Zonal, (b) Meridional average of climatology neutral surfaces’ distribution (1984-2013).

(b)

(a)


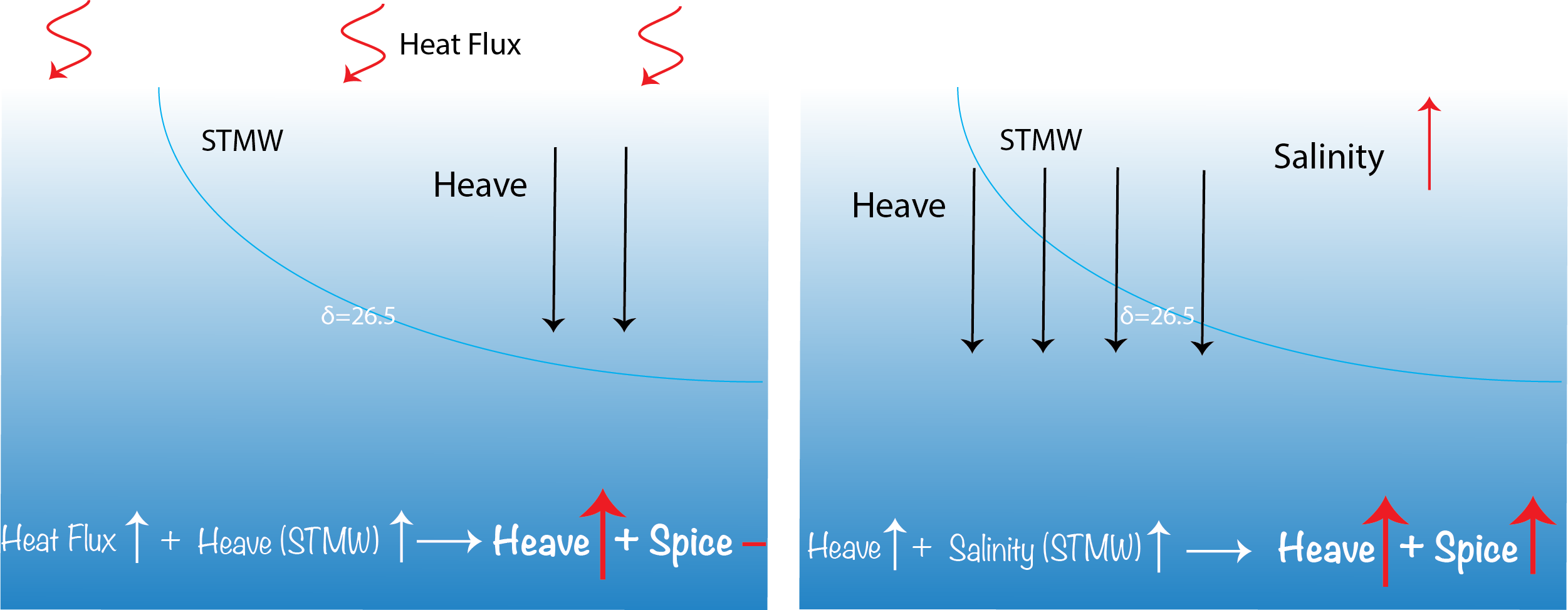


Fig. s5: Schematic of the warming mechanisms during (a) global surface warming acceleration and (b) slowdown.

|  | Corr | RMSE (x10^8^J/m^2^) | STD (x10^8^J/m^2^) | | Trend (x10^8^J/m^2^decade) | |
| --- | --- | --- | --- | --- | --- | --- |
|  |  | | ORAP5 | ORAS4 | ORAP5 | ORAS4 |
| 200m | 0.9711 | 2.12 | 5.76 | 5.44 | 1.61 | 1.49 |
| 700m | 8318 | 6.08 | 6.92 | 7.98 | 4.41 | 4.58 |
| 1500m | 8197 | 8.05 | 8.34 | 9.65 | 6.41 | 6.31 |

Table. s1: Quantitative comparison between monthly regional averaged OHC in the Agulhas Region computed from ORAP5 and ORAS4 reanalysis data, including correlation coefficient (Corr), root mean square error (RMSE), standard deviation (STD), and trend.
